# Inequalities in rural communities: adapting national deprivation indices for rural settings

**DOI:** 10.1093/pubmed/fdx048

**Published:** 2017-04-27

**Authors:** D Fecht, A Jones, T Hill, T Lindfield, R Thomson, A L Hansell, R Shukla

**Affiliations:** 1 UK Small Area Health Statistics Unit, MRC-PHE Centre for Environment and Health, Imperial College London, St Mary’s Campus, Norfolk Place, London, UK; 2 Norwich Medical School, University of East Anglia, Norwich Research Park, Norwich, UK; 3 Public Health for Lincolnshire, Lincolnshire County Council, Newland, Lincoln, UK; 4 Public Health Suffolk, Suffolk County Council, Endeavour House, 8 Russell Road, Ipswich, UK; 5 Public Health for Shropshire, Shropshire Council, Abbey Foregate, Shrewsbury, UK; 6 Imperial College Healthcare NHS Trust, London, UK; 7 Public Health England, 5 St Phillip’s Place, Birmingham, UK

**Keywords:** methods, public health, socioeconomics factors

## Abstract

**Background:**

Deprivation indices have been widely used in healthcare research and planning in the United Kingdom. Existing indices, however, are dominated by characteristics of urban populations that may be less relevant in capturing the nature of rural deprivation. We explore if deprivation indices can be modified to make them more sensitive to displaying rural disadvantage in England.

**Methods:**

The analysis focussed on the 2011 Carstairs Index (Carstairs2011) and the 2010 English Index of Multiple Deprivation (IMD2010). We removed all urban areas as identified by the Office for National Statistics Rural–Urban Area Classifications and mapped the Carstairs2011 and IMD2010 across the remaining rural areas using rural-specific quintiles.

**Results:**

Our method was effective in displaying much greater heterogeneity in rural areas than was apparent in the original indices. We received positive feedback from Directors of Public Health who confirmed that the observed patterns mirror their experiences and first-hand knowledge on the ground.

**Conclusions:**

Our maps of Carstairs2011 and IMD2010 for rural areas might strengthen the evidence base for rural planning and service provision. The modified deprivation indices, however, were not specifically formulated for rural populations and further work is needed to explore alternative input variables to produce a more rural-specific measure of deprivation.

## Introduction

Deprivation indices have been widely used in healthcare research and planning in the United Kingdom (UK) since the mid-1980s to target resources and services by local authorities and identify demand for healthcare.^1^ Similar indices have been developed and broadly employed in many other parts of the world, predominately European countries, Australia, New Zealand and Canada.^2–6^ These indices generally measure multiple components of material and social disadvantage to capture the multidimensional aspects of deprivation experienced by residents at small-area levels. Deprivation in this context not only relates to monetary poverty, but also follows Townsend’s definition of deprivation which is characterized by a general lack of housing, education, working and social conditions, amongst others.^7^

Early deprivation indices in the UK typically relied on data from population censuses. For example, the Carstairs Index,^8^ initially developed for Scotland, and now widely adapted internationally,^9,10^ is computed using input variables (unemployment, lack of car ownership, low occupational social class, overcrowding) from the census, and typically recorded for small geographical areas.

More recently, census-based indices have been largely superseded in the UK by national variants of the Index of Multiple Deprivation (IMD), which is the index predominantly used by the British Government. The IMD uses more than 30 input variables sourced from administrative databases.^11^ The IMD can therefore be updated more regularly; the first IMD for England, e.g. was published in 2000^12^ and subsequently updated in 2004, 2007, 2010 and most recently in 2015. These deprivation indices generally indicate a higher deprivation in urban areas, contrary to similar indices used in other countries, particularly the US and Canada.^13^

The various deprivation indices all provide a useful indication of which areas are more or less disadvantaged. They all suffer, however, from a number of limitations, in particular if deprivation in rural populations is of interest.^14,15^ This is because indices tend to focus on the type of material disadvantage that is most prevalent in urban populations, who constitute the majority. As such, urban areas tend to dominate the deprived end of index score distributions and the use of typical cut-offs such as quintiles identifies most rural areas as not deprived. A further challenge is that the nature of deprivation experienced by rural residents tends to differ from that experienced by their urban counterparts. Important aspects of rural deprivation relate to fuel poverty (i.e. households whose energy costs are higher than can be sustained by their income),^16^ hidden unemployment, and lack in opportunities such as poor access to services including shops and amenities, healthcare, childcare or digital services access.^17^ These disadvantages are not typically experienced by urban populations and hence are not considered in most deprivation indices. Because of the issue of urban dominance, standardizing these national indices across all small areas in the UK inevitable results in indicators being standardized around typical urban values.^18^

Because of their limitations in capturing rural disadvantage, evidence from both the UK and Canada suggests that deprivation indices generally capture health inequalities and healthcare needs better in urban areas than rural areas.^18–20^ These differences in deprivation between rural and urban areas may be driven by greater internal variability in deprivation within rural areas but little variation between rural areas, which suggests that the real variation is at the individual or household level.^20^ Deprivation indices, however, provide an aggregate measure of disadvantage for the whole population residing within the geographical area for which the index is calculated. Hence, the same score will be allocated to each individual and household within a given area and consequently variations in disadvantage within that area will not be detected. This is a particular problem for rural areas, in that deprivation tends to be present in small pockets (e.g. a few isolated houses on the edge of a village), but rural dissemination units are often very large.^21^

As a consequence of the limitations discussed, there is a danger that high levels of need and inequalities in rural areas can become overlooked when measuring health service needs using existing indices. There is a strong need therefore to apply deprivation indices in ways that will best reflect rural disadvantage. Here we explore how existing deprivation indices can be adapted to make them more sensitive to the identification of variation in rural deprivation and displaying rural disadvantage. We identified rural areas in England and re-standardized existing national deprivation indices to rural areas only.

## Methods

The analysis focusses on the 2011 Carstairs Index (Carstairs2011) and the 2010 English IMD (IMD2010). We computed Carstairs scores using data from the 2011 Census for all Census Output Areas (COAs) in England (2011 census boundaries; average population of 300 individuals) following the method developed by Carstairs and Morris.^8^ The Carstairs2011 includes the four variables: (i) unemployment—defined as unemployed males aged 16 and over as a proportion of all economically active males aged 16 and over; (ii) car ownership—defined as households which do not own a car; (iii) overcrowding—defined as households with one or more person per room; and (iv) low social class—defined as persons in households with an economically active head of household in a low social class.

The IMD2010 scores are produced for all lower layer super output areas (LSOAs) in England (2001 census boundaries; average population of 1500 individuals) and are accessible via the gov.uk website.^22^ The IMD2010 contains seven different domains of deprivation, all of which can be separated from the overall IMD. The domains relate to (i) income deprivation; (ii) employment deprivation; (iii) health deprivation and disability; (iv) education training and skills deprivation; (v) barriers to housing and services; (vi) crime; and (vii) living environment deprivation.^11^ Each domain contains several indicators (38 in total) that best capture the deprivation for that domain. To overcome problems associated with small numbers and large standard errors a shrinkage estimation is applied. Indicators are then combined to form the different domain scores. Domain scores are ranked and transformed to a specified exponential distribution. The domains are combined using weights to form an overall IMD. A comprehensive description of the methodology plus a list of all 38 indicators can be found in the English Indices of Deprivation 2010—Technical Report.^11^

We identified rural areas using the Office for National Statistics (ONS) Rural–Urban Area Classifications for 2011 (for COAs 2011) and for 2004 (for LSOAs 2001). We categorized areas as rural if they were classified in the Rural–Urban Area Classification as small town and fringes, villages and hamlets and isolated dwellings. All urban areas were removed from the analysis.

To create the Carstairs2011 for rural areas (*R*Car), we used the standard Carstairs calculation. We re-calculated the index scores and re-standardized rural areas only as follows:

$$R\mathrm{Car}=\sum_{i=1}^{n}\frac{X_{i}\;-\;\bar{x}}{{\mathrm{sd}}_{x}}$$

$$X_{i}=\frac{n_{i}}{d_{i}}$$

where *n*_*i*_ is the numerator of deprivation variable *X*_*i*_ (e.g. number of unemployed males), *d*_*i*_ is the denominator of deprivation variable *X*_*i*_ (e.g. number of economic active males), $\bar{x}$ is the mean of *X*_*i*_ and sd_*x*_ is the standard deviation of *X*_*i*_.

To map the Carstairs2011 for rural areas, we ranked the COAs in England according to their Carstairs score and categorized them into quintiles.

Due to the complexity of the IMD calculation (including shrinkage estimation in case of small numbers to move unstable scores towards the Local Authority average and factor analysis to combine indicators to domains), which does not allow a direct reproduction of the IMD, we could not re-standardize the IMD to rural areas only.^11^ To display the heterogeneity of the IMD2010 in rural areas, we instead mapped the IMD (and its domains) for rural LSOAs using quintiles of the IMD2010 specific to rural areas only.

For both Carstairs2011 and IMD2010 we used the Cohen’s kappa coefficient *K* to assess the magnitude of agreement between the original index and index for rural areas (expressed as quintiles).^23^*K* provides a direct measure of the probability of a COA or LSOA falling into the same deprivation quintile when applying the original index or the index for rural areas.

## Results

**Fig. 1:**
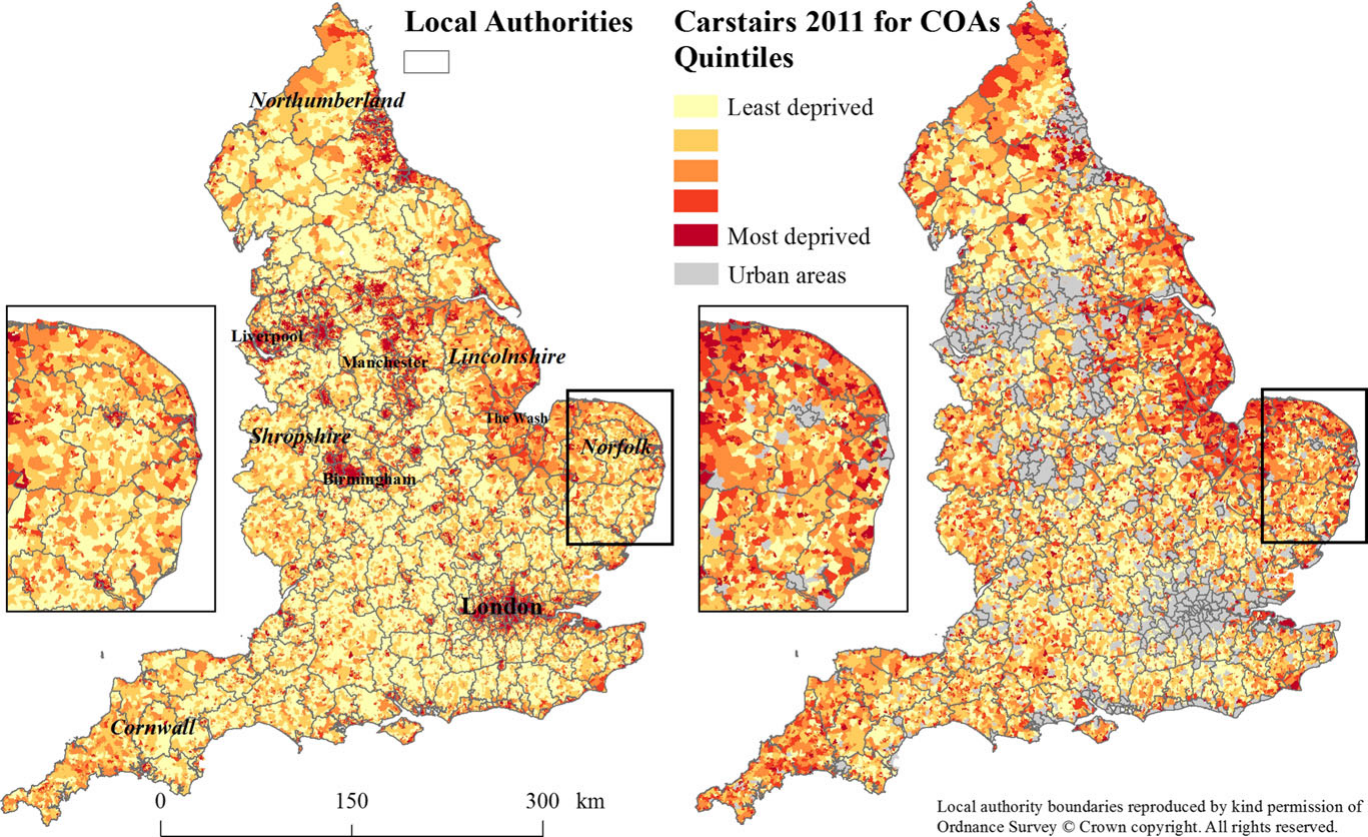
Carstairs2011 at census output area level (left) and Carstairs2011 for rural areas only (right).

**Fig. 2:**
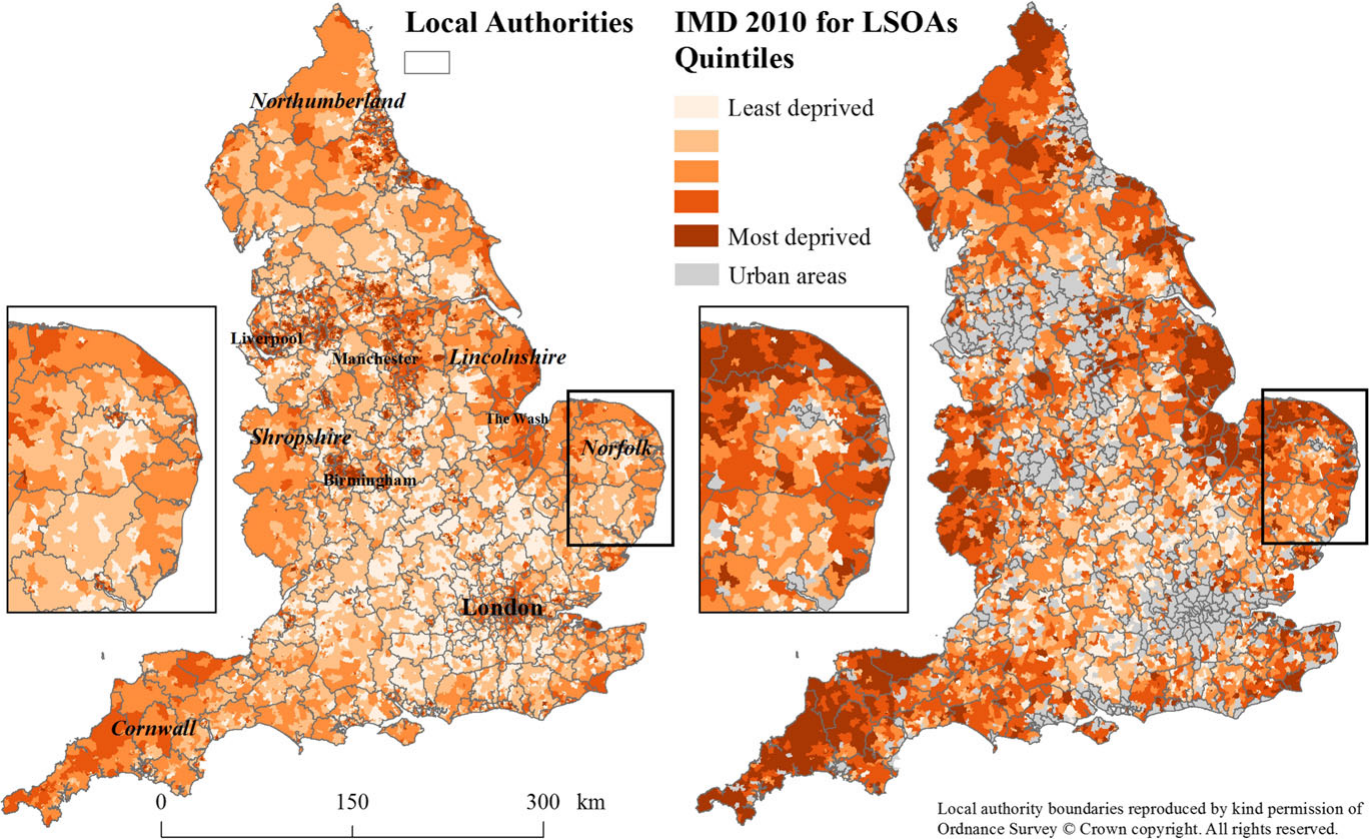
Index of Multiple Deprivation 2010 at lower layer super output area level (left) and Index of Multiple Deprivation 2010 for rural areas only (right).

We included 31 111 rural COAs (18%) and 6027 rural LSOAs (19%) in the analysis. As can be seen from Figs 1 and 2, both the Carstairs2011 and the IMD2010 for rural areas display much greater heterogeneity in mapped deprivation scores than was apparent in the original index calculations.

We observed areas of highest rural deprivation along the East coast of England, in particular, around The Wash in Lincolnshire and Norfolk; the border areas with Wales and Scotland, including Shropshire and Northumberland; and in Cornwall. In contrast, areas of lowest deprivation were similar in the original indices and those for rural areas and concentrated on the outskirts of major conurbations such as Greater London and the major cities of the Trans-Pennine region (Liverpool, Manchester, Sheffield, Leeds and Bradford).

Overall, deprivation patterns were similar between the Carstairs2011 for rural areas and the IMD2010 for rural areas. Due to differences in scale between smaller COAs (Carstairs2011) and larger LSOAs (IMD2010), however, differences at the local level could be observed. This was particularly apparent in Cornwall where the majority of LSOAs were in the most deprived quintile.

**Fig. 3:**
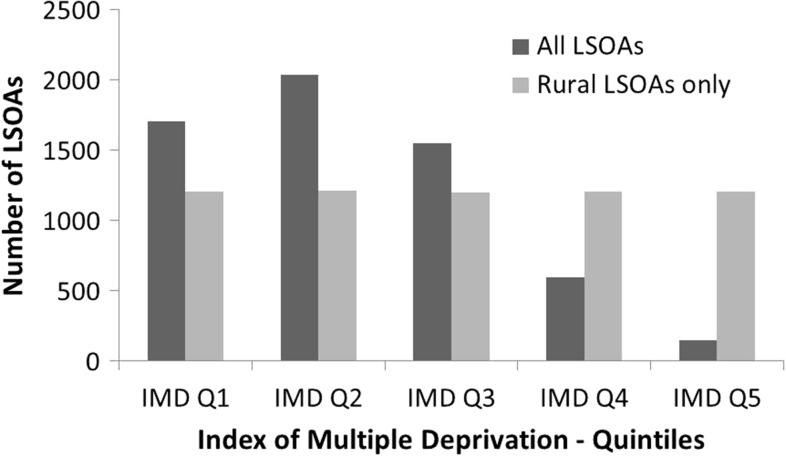
Number of lower layer super output areas (LSOAs) in rural areas categorized into Index of Multiple Deprivation quintiles (where Q1 represents LSOAs of low deprivation and Q5 LSOAs of high deprivation).

The distribution of deprivation quintiles in the original IMD2010 in comparison with the IMD2010 for rural areas demonstrated that a disproportionate number of rural areas (88%) were within the lowest three deprivation quintiles (Fig. 3). The kappa statistic further indicated very low overall agreement between the original index and index for rural areas: Carstairs2011, *K* = 0.07 and IMD2010, *K* = 0.18; both results were statistically significant (*P* < 0.001). The quintile accuracy was highest for least deprived quintile with 99% (Carstairs2011) and 100% (IMD2010) of COAs and LSOAs, respectively, falling within the first quintile for both original index and index for rural areas but low or very low for more deprived quintiles (Table 1). The majority of small areas were classified as either one quintile (52% for Carstairs2011, 56% for IMD2010) or two quintiles (22% for Carstairs2011, 10% for IMD2010) lower using the index for rural areas compared to the original index.

**Table 1:** Number of small areas (COAs for Carstairs2011, LSOAs for IMD2010) within each deprivation quintile using index for rural areas compared to original index

		Q 1	Q 2	Q 3	Q 4	Q 5	Total	Percentage
	*Original Carstairs2011*							
*Rural areas only Carstairs 11*	Q 1	6567	47	0	0	0	6614	99
	Q 2	5753	781	7	1	0	6542	12
	Q 3	1298	4806	104	1	0	6209	2
	Q 4	0	3162	2963	32	0	6157	1
	Q 5	0	0	2268	2649	672	5589	12
	Total	13618	8796	5342	2683	672	31111	
	*Original IMD2010*							
*Rural areas only IMD2010*	Q 1	1206	0	0	0	0	1206	100
	Q 2	499	710	0	0	0	1209	58
	Q 3	0	1201	0	0	0	1201	0
	Q 4	0	125	1081	0	0	1206	0
	Q 5	0	0	466	595	144	1205	12
	Total	1705	2036	1547	595	144	6027	

## Discussion

### Main findings of the study

In this research we modified existing deprivation indices, by re-standardization across rural areas only, with the purpose of highlighting rural deprivation. Our method was effective in displaying much greater heterogeneity in rural areas than was apparent in the original indices. Using the indices for rural areas only, the majority of areas were classified as either one or two deprivation quintiles more deprived than using the original indices. We identified previously hidden pockets of deprivation along the East coast of England, the Scottish border areas and Cornwall. The methodology employed to produce maps of the Carstairs2011 and IMD2010 for rural areas was developed in consultation with several Directors of Public Health (DsPH) working in rural areas. The maps received positive feedback from the DsPH who confirmed that the observed patterns correlated to their experiences and first-hand knowledge on the ground.

### What is already known on this subject

Overall health comparisons suggest that rural communities often experience better health than urban residents, the magnitude of differential disease rates depending on the specific health outcome under investigation.^24,25^ There are, however, persistent problems that put the focus on rural areas. Examples include the ageing population, road traffic accidents, fuel poverty and possibly excess winter deaths. In addition, the costs of providing services for rural residents may be considerably higher than for their urban counterparts.^26^ In England, public health funding has been historically allocated to local areas based on the principle of ‘equal access for equal need’.^27^ Based on this principle, the National Health Service (NHS) England allocates funding for healthcare to local areas based on their healthcare needs in a way that represents local areas’ fair share of available funding.^28^ The approach to assessing relative needs is based predominantly on the age profile of local populations, and also includes deprivation as measured by the IMD. Although age has been the dominant factor in determining the level of allocation to local authorities, deprivation is also driving funding differences between areas.^29^ It is of concern that the higher cost of healthcare provision in rural areas, coupled with the inability of the IMD to appropriately capture rural deprivation, raises the potential for serious underfunding of rural healthcare needs.^30^ The particular challenges of identifying deprivation and needs within rural communities will become even more important with the planned move to business rate retention by Local Authorities in 2020, when locally raised business rates will be the sole basis for funding for local services including public health and care services.

A number of limitations of currently used area-based indices for rural settings, therefore, still exist. These include their greater focus on material over social deprivation, whilst social deprivation such as isolation, both physical and social, may be more relevant to rural areas. In addition, standardization of indices for larger area levels can lead to urban bias, with more dispersed and isolated rural disadvantage being averaged out by wider more affluent neighbouring zones. It would appear that there remains the need for further work to identify a set of indicators which may be used to better identify rural deprivation and the inequalities within rural communities. Such indicators might focus on households in fuel poverty, travel time to services and adults and children in need of social care.

### What this study adds

This is the first stage in a programme of work that aims to understand how the challenges of rural health and care provision may be better addressed. The work presented here provides a straightforward approach to identifying variation in deprivation within rural areas using existing national indices. Such variation is easily missed if using universal measures across urban and rural areas. This is of high importance to policy and decision-making activities and could lead to biased formulation of programs and funding allocation schemes. Highlighting rural deprivation therefore helps decrease the health inequality gap between rural and urban areas and consequently reducing the burden on the NHS. Our maps of the Carstairs2011 and IMD2010 for rural area form a new evidence base for rural health and planning in areas with a strong element of need. For example, our maps are being used by DsPH as part of the local refresh of Joint Strategic Needs Assessment (JSNA) and have provided a reference point to ‘rural proof’ local decision making. The JSNA provides the evidence for identifying the priorities in each area’s Joint Health and Wellbeing Strategy and these maps will allow DsPH to ensure that the priorities take account of rural deprivation and target funding towards areas affected more specifically. The maps have also been used by a Local Enterprise Partnership (LEP). LEPs are the channel for large amounts of financial resources for economic growth and skills programmes. There is a tendency to direct resources to places with existing high levels of economic activity but a clear demonstration of rural deprivation allows scope for raising skill levels and creating jobs where they are scarce.

The underlying data as well as all maps, including the IMD domains for rural areas can be downloaded via the website: http://www.sahsu.org/content/data-download.

### Limitations of the study

Our approach to remove urban areas and re-standardize across rural areas means that the indices still contain the same input variables as originally used. As such, the rural indices produced do not capture additional dimensions of rural disadvantage that were not contained in the original indices, but rather highlight previously hidden variations in rural disadvantage. The Carstairs index, e.g. includes a variable on non-car ownership which is a much poorer measure of rural than it is for urban disadvantage. Rural households might own a car but lack the means to keep it operational which, due to a scarcity of alternative transport sources, prevents access to services and contribution to social life.

The importance of car-ownership and accessibility in general however will depend on the population density and size of the small area.^31^ An associated limitation is that in the IMD calculation a shrinkage estimation is used to move LSOA scores of areas with small population counts (and large standard errors) towards the more robust Local Authority mean.^11^ This could potentially distort the IMD2010 for rural areas in areas close to urban centres. A further limitation is that we were limited to the same spatial resolution as the data used to produce the original indices due to many input data not being available at other resolutions. Work by Huby *et al.*^21^ has demonstrated how changes in the size of spatial units influences the magnitude of measures of inequality in poverty in rural areas. Our maps suffer from the same limitation as the original indices in that they may not fully capture the fragmented and often very local nature of rural material deprivation. Further work is also needed to explore the differences in national level funding allocation if using rural specific or urban specific indices of deprivation as a gauge to formulate the allocation and the impact of weighting of factors such as age and deprivation.

## Funding

The work of the UK Small Area Health Statistics Unit is funded by Public Health England as part of the MRC-PHE Centre for Environment and Health, funded also by the UK Medical Research Council.

## Authorship

DF: conception and design of the study; data acquisition, analysis and interpretation; drafting the article. AJ: conception and design of the study; interpretation of results; drafting the article. TH: conception of study; revising the article. TL: conception of study; revising the article. RT: conception of study; revising the article. AH: conception of study; revising the article. RS: conception of study; revision of article.
